# Glucocorticoid trajectories in preterm infants—born too soon, wired differently

**DOI:** 10.1210/clinem/dgag048

**Published:** 2026-02-14

**Authors:** Tanja Kuiri-Hänninen, Christa Flück, Sanna Silvennoinen, Michael Groessl, Ulla Sankilampi

**Affiliations:** Department of Pediatrics, Kuopio University Hospital, 70210 Kuopio, Finland; Kuopio Pediatric Research Unit (KuPRU), University of Eastern Finland, 70210 Kuopio, Finland; Pediatric Endocrinology, Diabetology and Metabolism, Department of Pediatrics, Inselspital Bern University Hospital, University of Bern, 3010 Bern, Switzerland; Department of Biomedical Research, University Hospital Inselspital, University of Bern, 3010 Bern, Switzerland; Department of Pediatrics, Kuopio University Hospital, 70210 Kuopio, Finland; Department of Biomedical Research, University Hospital Inselspital, University of Bern, 3010 Bern, Switzerland; Department of Nephrology and Hypertension, University Hospital Inselspital, University of Bern, 3010 Bern, Switzerland; Department of Pediatrics, Kuopio University Hospital, 70210 Kuopio, Finland; Kuopio Pediatric Research Unit (KuPRU), University of Eastern Finland, 70210 Kuopio, Finland

**Keywords:** prematurity, glucocorticoid, fetal adrenal cortex, GC-MS, fetal-neonatal transition

## Abstract

**Context:**

Levels of glucocorticoid (GC) precursors are elevated in preterm infants, whereas clinical signs of GC deficiency are frequently observed in neonatal intensive care units.

**Objective:**

To describe the maturation of the GC metabolic pathway in preterm infants during the first year of life.

**Design and Setting:**

Spot urinary samples (n = 154) were collected in the neonatal intensive care unit and at follow-up visits.

**Participants:**

Sixteen preterm infants (8 boys) born <30 weeks of gestational age. Data of full-term infants were available from the same laboratory.

**Main Outcome Measures:**

Urinary levels of GC precursor metabolites and 13 GC metabolites were quantitated by gas chromatography-mass spectrometry. Enzyme activities were calculated by product/substrate ratios. Mixed models were used for statistical analyses.

**Results:**

The levels of GC precursors remained high in preterm infants until term-equivalent age (TEA), after which they decreased (*P* < .001). However, GC production, estimated by the sum of 13 GC metabolites did not change significantly over time in preterm infants and was higher in preterm than in full-term infants after 1 week of age (*P* = .044 −<.001). The sum of 13 GC metabolites/tetrahydro-11-deoxycortisol ratio (representing CYP11B1 activity) increased in preterm infants after TEA (*P* < .001), whereas the 16α-OH-DHEA/5-PT ratio (representing 17,20-lyase activity) decreased (*P* < .001). The ratio of cortisone metabolites to cortisol metabolites was higher in preterm infants before TEA than thereafter (*P* < .001).

**Conclusion:**

Despite a high production rate of GC precursors in preterm infants, the total GC production remained relatively constant and was regulated at the level of CYP11B1. Persisting differences between preterm and full-term infants in GC precursor levels as well as in GC production were observed, indicating possible programming effects of preterm birth.

Glucocorticoids (GCs) are essential for fetal organ maturation in late pregnancy and promote survival after birth (1, 2). Unlike the adult adrenal cortex, which is divided into 3 distinct zones, the fetal cortex initially comprises only 2 zones: the fetal and the definitive. The fetal zone exhibits CYP11A1 and CYP17A1 enzyme activities but lacks HSD3B2, the enzyme necessary for de novo GC synthesis (3). Throughout pregnancy, the main product of the fetal zone is dehydroepiandrosterone sulfate (DHEAS), which serves as a precursor for placental estrogen synthesis. GC synthesis occurs in the transitional zone, which forms between the fetal and definitive zones and begins expressing HSD3B2 around midgestation (3-5). In addition to CYP17A1 and HSD3B2, the enzymes CYP21A2 and CYP11B1 are also required for GC production. Besides the fetal hypothalamic-pituitary axis, the placenta also produces ACTH and corticotropin-releasing hormone, hormones that stimulate adrenal steroidogenesis, with corticotropin-releasing hormone levels increasing, especially in late pregnancy (6, 7). ACTH receptors (MC2R) are expressed in the fetal adrenal cortex already during the first trimester (8, 9). Consequently, the integrated function of the fetoplacental unit results in progressive increase of both estrogen and GC production in the last trimester, preparing the fetus for transition to the extrauterine life (10, 11).

In preterm (PT) birth, the function of the fetoplacental unit is disrupted prematurely. Survival and health outcomes of PT babies have significantly improved through antenatal exposure to synthetic GCs administered to the mother, a practice that has been evidence-based in perinatal care for decades (12). Despite these advancements, extremely PT infants often display signs of GC deficiency during the first weeks of life, such as hypoglycemia and vasoactive-resistant hypotension, which improves with GC therapy regardless of cortisol levels (reviewed in (13-15)). These signs are attributed to the putative immaturity of their hypothalamic-pituitary-adrenal (HPA) axis and its inadequate response to stress (14, 16-18). Studies suggest that this relative adrenal insufficiency is associated with an increased risk of subsequent bronchopulmonary dysplasia (BPD), a chronic inflammatory lung disease of prematurity, which is linked to poor growth, adverse neurodevelopmental outcomes, and increased mortality (19, 20). Postnatal serum cortisol levels are influenced by gestational age and may not adequately reflect the adrenal stress response, complicating the diagnosis of adrenal insufficiency in PT infants (16, 21-25). Nevertheless, postnatal systemic GC therapy is commonly used among extremely PT infants for cardiorespiratory instability or to prevent and treat BPD during the first weeks of life (14, 26)

In PT infants, the postnatal adrenal steroid production differs from that of full-term (FT) infants. Despite the disruption of the fetoplacental unit function at birth, the fetal zone activity of the adrenal cortex continues until term-equivalent age (TEA), indicated by elevated DHEAS and other fetal zone metabolites in their serum and urine (27-31). The mechanisms maintaining fetal zone activity until TEA and the effects of high fetal zone steroid levels in PT infants are not well understood. Elevated levels of 17-hydroxyprogesterone (17-OHP), a GC precursor, are also observed in PT infants, similar to newborns with congenital adrenal hyperplasia resulting from defects in HSD3B2, CYP21A2, or CYP11B1 (24, 25, 32). Lower activity of the CYP11B1 enzyme, crucial for GC synthesis, has been suggested in PT babies compared to FT babies (25, 32), potentially explaining the accumulation of 17-OHP and limiting GC production. Higher GC precursor levels have been associated with the risk of developing BPD (20). GC action is further modulated by tissue-specific expression of enzymes such as HSD11β2 (inactivates cortisol to cortisone), HSD11β1 (reactivates cortisone to cortisol) (33), and 5α- and 5β-reductases (34), which irreversibly metabolize GCs for excretion.

The GC biosynthetic pathway is clearly important in PT infants; however, postnatal changes in the metabolism and excretion of GCs in these infants remain poorly understood. The aim of the present study was to examine longitudinal changes in GC metabolism during the first year of life in infants born PT at less than 30 weeks of gestation. Specifically, we aimed to describe changes in GC precursor and metabolite levels, measured through urinary steroid profiling, and their association with the prolonged secretion of the fetal zone metabolite 16α-OH-DHEA in PT infants in comparison to FT infants. In addition, to evaluate maturational changes over time, enzyme activities in the GC pathway were estimated longitudinally by product/substrate ratios.

## Material and methods

A total of 154 serial spot urine samples were collected from 16 PT infants born at less than 30 weeks of gestation (boys n = 8), starting from 1 week of age and continuing up to 18 months after birth.

Antenatal GC treatment (intramuscular betamethasone 12 mg in 2 doses 24 hours apart) was given to all mothers of PT infants. Two mothers received only a single dose of antenatal GC. The median time between antenatal GC administration (first dose) and delivery was 8 days in boys (range, 0-10 days) and 13 days in girls (range, 2-41 days). Characteristics of the PT infants are presented in Table 1. All girls and 5 boys were born by the cesarean section. The study included 1 pair of twin boys and 1 pair of twin girls. All but 2 boys had respiratory distress syndrome and required surfactant treatment. Birth weight and length were converted to SD scores using the population-based birth size reference for singletons or twins (35). Necrotizing enterocolitis was defined as Bell stage II or III (36). Intraventricular hemorrhage was diagnosed with ultrasonography using Papile's classification (37). BPD was diagnosed at 36 postmenstrual weeks using the oxygen reduction test (38).

**Table 1:** Characteristics of the PT infants

	Boys n = 8		Girls n = 8	
Gestational age (weeks)	27.0	23.4-29.7	27.0	24.0-29.4
Cesarean section	3		8	
Birth weight (grams)	1100	540-1630	710	475-1360
Birth weight (SDS)	0.97	−0.96 to 2.77	−1.27	−3.69 to 0.50
Birth length (cm)	35.8	30.0-40.5	33.5	28.0-38.0
Birth length (SDS)	0.69	−0.72 to 2.57	−1.50	−3.38 to −0.20
Apgar 5 minutes	7	2-9	7	2-9
Sepsis	2		2	
Meningitis	0		1	
NEC	1		1	
RDS	6		8	
BPD	3		4	
IVH				
Grade I-II	1		3	
Grade III-IV	0		2	
ROP	1		3	

Abbreviations: NEC, necrotizing enterocolitis; ROP, retinopathy of prematurity.

Data are presented as median and range or number of patients.

Birth weight and length were converted to SD scores using the population-based birth size reference for singletons or twins (35). NEC was defined as Bell stage II or III (36). Intraventricular hemorrhage (IVH) was diagnosed with ultrasonography using Papile's classification (37). Bronchopulmonary dysplasia (BPD) was diagnosed at 36 postmenstrual weeks using the oxygen reduction test (38).

The number of samples collected per infant ranged from 4 to 15 (median, 9). Ninety-five of 154 samples (62%) were collected by TEA. The sampling times according to postmenstrual age (PMA; sum of gestational age and chronological age) are presented in Fig. S1 (39).

Three boys and 5 girls received postnatal GC treatment (see Fig. S1 (39) for details). The GC treatment at the time of sampling (n = 13, Fig. S1 (39)) was considered in the statistical analyses.

Samples were collected after a written informed consent was obtained from the parents. The ethics committee of the Northern Savo hospital district, Finland, approved the study (permission numbers 11/2008 and 417/2015).

**Figure 1:**
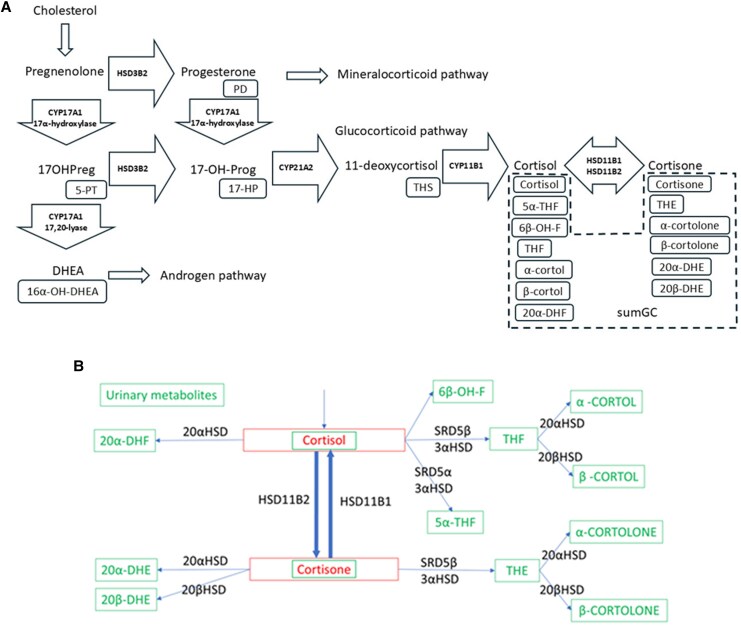
(A) A simplified illustration of the steroid pathways relevant to this study. Urinary metabolites are presented in boxes, and steroid metabolizing enzymes inside arrows. GC metabolites summarized for the analyses (sumGC) are in a box marked with a dashed line. (B) Urinary metabolites (inside boxes) of cortisol and cortisone and enzymatic pathways relevant to this study.

Urinary levels of GC precursor metabolites pregnanetriol (5-PT), pregnanediol (PD), 17-hydroxypregnanolone (17-HP), and tetrahydro-11-deoxycortisol (THS), as well as the fetal zone metabolite 16α-OH-DHEA were quantified. Additionally, 13 urinary GC metabolites were quantitated: 7 metabolites of cortisol (cortisol, 6β-hydroxycortisol [6β-OH-F], tetrahydro-11-deoxycortisol [THF], 5α-tetrahydrocortisol [5α-THF], α-cortol, β-cortol, 20α-dihydrocortisol), and 6 metabolites of cortisone (cortisone, tetrahydrocortisone, α-cortolone, β-cortolone, 20α-dihydrocortisone, 20β-dihydrocortisone). A list of all steroids analyzed in this study, including systematic names, is given in Table S1 (39). For the analyses, the sum of these 13 GC metabolites was calculated (sumGC). A simplified illustration of the steroid pathways relevant to this study are depicted in Fig. 1A and 1B.

To evaluate the enzyme activities on the GC biosynthetic and metabolic pathways, the ratios of urinary metabolites on each side of steroidogenic and GC metabolizing enzymes were calculated (product/substrate ratios): 16α-OH-DHEA/5-PT (17,20-lyase activity of CYP17A1 before HSD3B), 17-HP/5-PT (3βHSD activity), 17-HP/PD (17α-hydroxylase activity of CYP17A1 after HSD3B), THS/17-HP (CYP21A2 activity), sumGC/THS (CYP11B1 activity), 5α-THF/cortisol (SRD5A activity), THF/cortisol (SRD5B activity), THE/cortisone (SRD5B activity), and sum of all cortisone metabolites/sum of all cortisol metabolites (relative HSD11B activity).

Data of FT babies were obtained from a previous longitudinal study (the Bern Baby Urine study), which investigated 43 healthy infants (22 females) born at 37 gestational weeks or later during the first year of life at 13 time points (from the chronological age of 1 week to 49 weeks) (40-42).

Steroids were quantified using gas chromatography-mass spectrometry following an established method (43). In brief, 1.5 mL of urine was spiked with a mixture of isotopically labeled internal standards covering all steroid classes, followed by solid phase extraction, enzymatic hydrolysis, derivatization (methoxamine and N-trimethylsilyl-imidazole), and purification using liquid-liquid extraction with using cyclohexane and water. All measurements were performed on a 7890A gas chromatograph coupled to a mass selective detector (5977; both Agilent Technologies, USA). Steroid concentrations were normalized to the creatinine level of the corresponding sample, as measured by the QuantiChrom Creatinine Assay (DICT-500; BioAssay Systems, USA).

### Statistics

Groups were compared by using linear mixed models analysis (IBM SPSS Statistics for Windows, version 27 [IBM Corp., Armonk, NY, USA]). Comparisons between PT and FT groups were performed both according to chronological age (at 1, 3, and 5 weeks after birth), to evaluate the changes in urinary GC levels after birth and cessation of the placental hormone supply, and according to postmenstrual age (PMA) to account for the immaturity of the PT babies and to evaluate maturation of steroid pathways. For comparisons according to PMA, the data were categorized in 8 PMA categories: <30 weeks of PMA, 30 to 33 weeks of PMA, 34 to 37 weeks of PMA, TEA (38-41 weeks of PMA), 0 to 2.9 months of corrected age (CA) (42-51 weeks of PMA), 3 to 5.9 months of CA (52-63 weeks of PMA), 6 to 9.9 months of CA (64-77 weeks of PMA), and ≥10 months of CA (78-99 weeks of PMA). In FT babies, week 1 samples were included in PMA category TEA. In the mixed models analysis, subject and twinness were included as random effects while group (PT/FT), sex, time point/PMA category and sampling during the postnatal GC treatment (yes/no) were included as fixed effects.

## Results

There was no sex difference in any of the measured metabolite levels in PT infants (see Table 1 for patient characteristics); therefore, the results of boys and girls were analyzed together. Because PMA was a strong determinant of changes in GC precursor and GC metabolite levels in PT infants, the results are first presented according to PMA. Second, to evaluate the changes in GC metabolism in relation to birth and cessation of the placental hormone supply, results at 1, 3, and 5 weeks after birth were compared between PT and FT infants and are given in Figs. S2 and S3 (39).

### Urinary 16α-OH-DHEA and GC precursor levels in PT and FT infants by PMA

**Figure 2:**
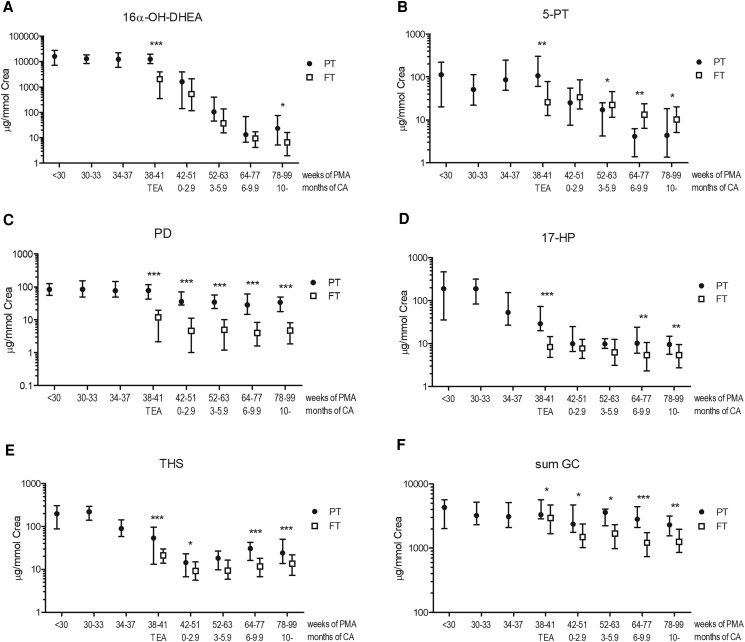
Median levels with 25th and 75th percentiles of (A) 16α-OH-DHEA (the main fetal zone metabolite), (B) 5-PT (pregnentriol, 17α-hydroxypregnenolone metabolite), (C) PD (pregnanediol, progesterone metabolite), (D) 17-HP (17-hydroxypregnenolone, 17α-hydroxyprogesterone metabolite), (E) THS (tetrahydro-11-deoxycortisol, 11-deoxycortisol metabolite), and (F) sum GC (sum of 13 GC metabolites) in postmenstrual age categories in preterm (PT) infants and full-term (FT) infants. Note logarithmic scale. ***P < .001, **P < .01, *P < .05 for the difference between PT and FT in the mixed models analysis. CA, corrected age (starting from TEA); PMA, postmenstrual age (sum of gestational age and chronological age in weeks); TEA, term-equivalent age.

Levels of the main urinary fetal zone metabolite, 16α-OH-DHEA remained high in PT infants until TEA, when the median levels in PT infants were almost 8-fold higher than FT infants (*P* < .001). Thereafter, 16α-OH-DHEA levels decreased with increasing PMA in both groups (*P* < .001 for the decrease) (Fig. 2A).

5-PT (a metabolite of 17-hydroxypregnenolone [17-OHPreg]), which is a precursor to both DHEAS and 17-OHP, had high levels in PT infants until TEA and then decreased (*P* < .001), following the same pattern as 16α-OH-DHEA (Fig. 2B). PD (a metabolite of another 17-OHP precursor progesterone) levels did not change significantly in PT infants and remained higher than in FT infants until the end of the follow-up (*P* < .001) (Fig. 2C).

Levels of 17-HP (a metabolite of 17-OHP) and THS (a metabolite of 11-deoxycortisol, the closest precursor to cortisol) were initially high in PT infants and started to decline after 30 to 33 weeks of PMA (Fig. 2D and 2E). At TEA, both levels were higher in PT infants (*P* < .001) but then decreased and came closer to the levels of FT infants. However, from 64 to 77 weeks of PMA (6-9 months of CA), both were again significantly higher in PT infants.

### Urinary GC metabolite levels in PT and FT infants by PMA

Although the levels of individual GC precursor hormone metabolites, as well as 16α-OH-DHEA, changed significantly with increasing PMA, the sumGC remained quite constant in PT infants throughout the first year of life (no statistically significant change between PMA categories). Notably, the sumGC level was higher in PT infants compared to FT infants at all PMA categories (*P* = .041- < .001, Fig. 2F).

**Figure 3:**
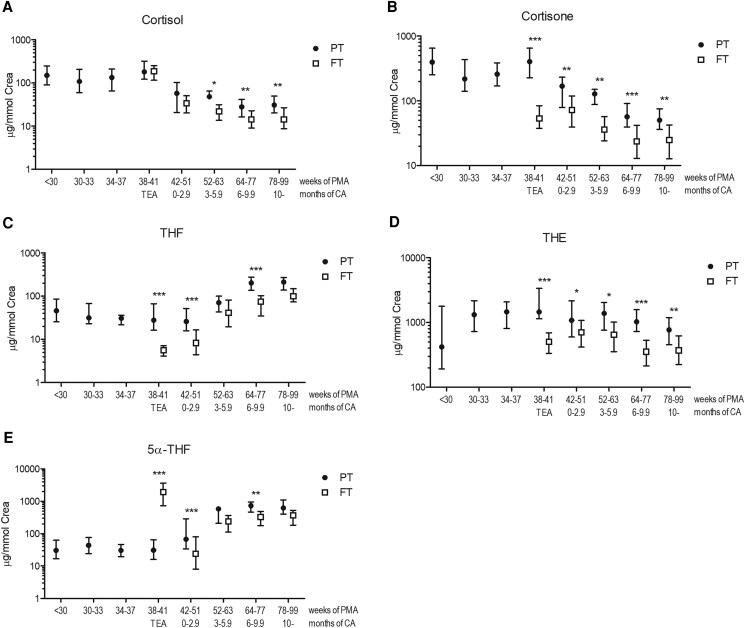
(A-E) Secretion pattern of 5 key urinary GC metabolites (median levels with 25th and 75th percentiles) in preterm (PT) and full-term (FT) infants in the postmenstrual age categories. THE, tetrahydrocortisone; THF, tetrahydrocortisol; 5α-THF, 5α-tetrahydrocortisol. Note logarithmic scale. ***P < .001, **P < .01, *P < .05 for the difference between PT and FT in the mixed models analysis. CA, corrected age (starting from TEA); PMA, postmenstrual age (sum of gestational age and chronological age in weeks); TEA, term-equivalent age.

There were significant changes in the levels of individual GC metabolites over time (Fig. 3A-3E). Most of the GC metabolite levels were higher before TEA, and decreased significantly thereafter (cortisol, cortisone, 6β-OH-F, α-cortol, β-cortolone, 20α-dihydrocortisol, 20α-dihydrocortisone, 20β-dihydrocortisone). However, 5α-THF and THF were low before TEA (<2% of sumGC) and then increased significantly (*P* < .001), 5α-THF forming >25% of sumGC after 52 to 63 weeks of PMA. THE was the main metabolite in PT infants after 30 weeks of PMA (33%-50% of sumGC). Although there were significant differences in the GC metabolite levels between FT and PT groups, the pattern of changes after TEA was similar in both groups (Fig. 3A-3E).

### Maturation of GC biosynthetic and metabolic pathways depicted by calculated enzyme activities

To evaluate the maturational changes of the enzymatic activities in the GC and fetal zone pathways, the ratios of urinary precursor to product catalysis of steroidogenic enzymes were calculated.

**Figure 4:**
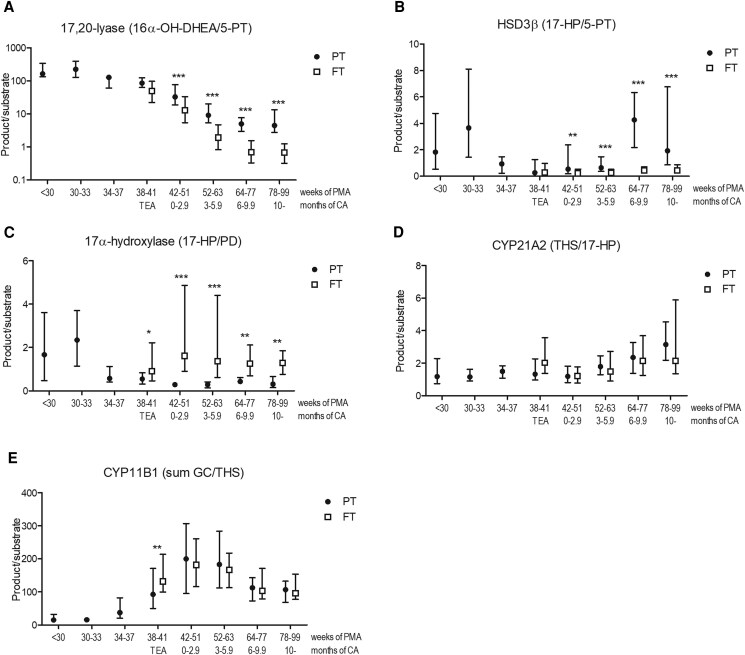
(A-E) Longitudinal calculated enzyme activities (median with 25th and 75th percentiles) in fetal zone and GC pathways in preterm (PT) and full-term (FT) infants in postmenstrual age (PMA) categories. Note logarithmic scale in panel A. 5-PT, pregnenetriol; 17-HP, 17-hydroxypregnenolone; PD, pregnanediol; THS, tetrahydro-11-deoxycortisol; sum GC, sum of 13 GC metabolites. ***P < .001, **P < .01, *P < .05 for the difference between PT and FT in the mixed models analysis. CA, corrected age (starting from TEA); PMA, postmenstrual age (sum of gestational age and chronological age in weeks); TEA, term-equivalent age.

17,20-lyase activity was very high before TEA in PT infants (Fig. 4A). At TEA, the groups did not differ significantly, but after TEA the enzyme activity was significantly higher in PT infants, although decreasing in both groups (*P* < .001). 3βHSD activity was elevated in PT infants before TEA, decreased to similar level as in FT infants at TEA, but then increased again above FT levels (*P* < .001, Fig. 4B). 17α-hydroxylase activity was highest before 34 weeks of PMA, then decreased, and from TEA was significantly lower in PT than in FT infants (*P* < .001, Fig. 4C). CYP21A2 activity did not differ significantly between the groups; it remained constant until 52 to 63 weeks of PMA (3-6 months of corrected age) and then increased in both groups (Fig. 4D). In PT infants, CYP11B1 activity was low before 34 weeks of PMA, then increased, and after TEA, was similar to FT infants (Fig. 4E). The activities of 17,20-lyase and CYP11B1 changed reciprocally over time in PT infants, with equal activities at TEA.

**Figure 5:**
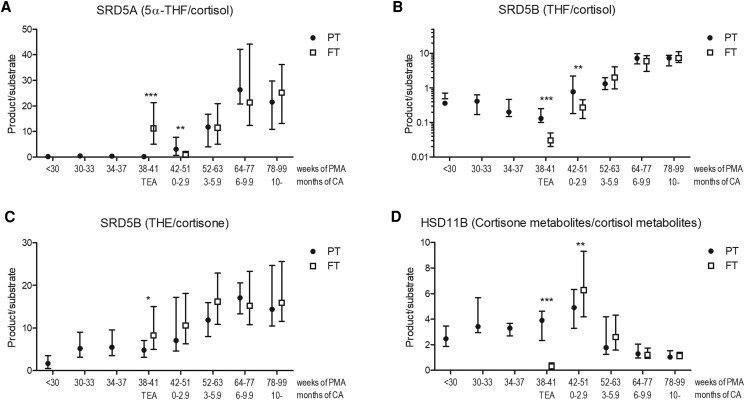
(A-D) Longitudinal calculated enzyme activities (median with 25th and 75th percentiles) of GC metabolizing enzymes in preterm (PT) and full-term (FT) infants in postmenstrual age (PMA) categories. Note logarithmic scale in panel B. ***P < .001, **P < .01, *P < .05 for the difference between PT and FT in the mixed models analysis. 5α-THF, 5α-tetrahydrocortisol; CA, corrected age (starting from TEA); PMA, postmenstrual age (sum of gestational age and chronological age in weeks); TEA, term-equivalent age; THE, tetrahydrocortisone; THF, tetrahydrocortisol.

GC metabolism through SRD5A activity was low in PT infants until TEA, then increased (*P* < .001) and was similar to FT infants after 42 to 51 weeks of PMA (0-2 months of corrected age) (Fig. 5A). THE/cortisone ratio was higher than THF/cortisol ratio reflecting higher relative activity of SRD5B on cortisone than cortisol, whereas both increased over time (Fig. 5B and 5C).

The ratio of cortisone metabolites to cortisol metabolites depicting relative HSD11B1/2 activities was low in FT infants at TEA (median ratio, 0.3; 13-fold lower than in PT infants; *P* < .001; Fig. 5D). By 42 to 51 weeks of PMA (0-2 months of corrected age) the ratio increased in FT infants (*P* < .001) to a higher level than in PT infants (*P* = .009). In PT infants, this ratio varied from 2.5 to 4.9 until 42 to 51 weeks of PMA (0-2 months of corrected age) and then decreased (*P* < .001) close to 1 by the end of the first year, similar to FT infants. This indicates that up to 42 to 51 weeks of PMA (0-2 months of corrected age), GCs are predominantly inactivated to cortisone metabolites in PT infants (higher relative HSD11B2 than HSD11B1 activity), whereas FT infants appear to have transient high HSD11B1 activity right after birth.

### Urinary 16α-OH-DHEA, GC precursor, and metabolite levels at 1, 3, and 5 weeks after birth

16α-OH-DHEA levels and other GC precursor levels, except 5-PT, were higher in PT than in FT infants from 1 to 5 weeks of age (*P* < .001 for all, Fig. S2A-S2E (39)). Levels of 16α-OH-DHEA, 5-PT, and PD did not change significantly in either group from 1 to 5 weeks of age. 17-HP and THS levels increased in PT infants from 1 to 5 weeks of age (*P* < .001 and *P* = .004, respectively). THS decreased in FT infants from 1 to 5 weeks of age (*P* < .001).

At 1 week of age, sumGC was similar in FT and PT infants (*P* = .7, Fig. S2F (39)), but the proportions of GC metabolites were very different between the groups. In FT infants, 5α-THF was the major metabolite comprising more than two thirds of the whole sumGC, also THE levels were significantly higher than in the PT infants (*P* = .034). All other GC metabolites were significantly higher in PT than in FT infants (*P* < .001 for all). SumGC decreased from 1 to 3 weeks of age (*P* < .001) in FT infants and at 3 and 5 weeks of age, sumGC was higher in PT than in FT infants (*P* < .001, Fig. S2F (39)).

### Calculated enzyme activities at 1, 3, and 5 weeks after birth

HSD3B and 17,20-lyase activities were higher and CYP11B1 activity lower in PT than in FT infants (*P* < .001 at all time points, Fig. S3A and S3C, and S3E (39)). No differences were observed in 17α-hydroxylase and CYP21A2 activities between PT and FT infants (Fig. S3B and S3D (39)).

The median SRD5A activity was 100-fold higher in FT infants in comparison to PT infants at 1 week of age but decreased by 3 weeks of age and was then 2-fold lower than in PT infants (*P* = .035, Fig. S3F (39)). In PT infants, SRD5A activity did not change and by 5 weeks of age, activity was similar between the groups. In FT infants, the median relative HSD11B activity increased from 0.3 at 1 week to 7.8 at 3 weeks of age (*P* < .001, Fig. S3G (39)). In PT infants this ratio increased from 2.1 at 1 week of age to 3.5 at 5 weeks of age (*P* = .005).

## Discussion

Glucocorticoids are essential for postnatal viability, playing a critical role in regulating energy metabolism, cardiovascular stability, lung function, and thermogenesis. In newborns, cortisol deficiency may manifest as life-threatening hypotension, hypoglycemia, dehydration, vomiting, and shock. When mineralocorticoid deficiency is also present, hyponatremia and hyperkalemia may occur. Adrenal insufficiency in the neonatal period is rare and, in addition to genetic disorders, may result from infections, adrenal hemorrhage, or iatrogenic causes (44). The term *relative adrenal insufficiency* refers to a clinical condition characterized by signs of adrenal insufficiency (typically cardiovascular instability) in which cortisol production is present but inadequate for the degree of stress and administration of GCs typically leads to clinical improvement within hours (reviewed in (14, 15, 18)). Relative adrenal insufficiency in extremely preterm infants (also known as transient adrenal insufficiency of prematurity) has been described since the 1990s. However, because its pathophysiology remains poorly understood, no definitive diagnostic criteria exist. Consequently, GC therapy is often initiated based on clinical suspicion and continued or discontinued according to the clinical response (14, 15, 18) Systemic GCs are commonly used to prevent and treat BPD, and an association between BPD and relative adrenal insufficiency has been suggested (19, 20). Systemic GC therapy in PT infants has been also linked to significant short- and long-term risks, including increased susceptibility to infections, gastrointestinal and endocrine complications, as well as adverse neurodevelopmental and behavioral outcomes, which may be dependent on developmental maturity and postnatal age (14, 15, 26). To guide the appropriate, effective and safe use of systemic GCs in PT infants, a comprehensive understanding of the complexity of GC biosynthesis and metabolism in this population is essential.

In this study, we describe the maturation of GC biosynthesis and metabolism in PT infants born <30 weeks of gestation in comparison to FT infants during the first year of life through longitudinal urinary steroid profiles. We found that overall GC production estimated by the sum of 13 urinary GC metabolites was not compromised in PT infants and was regulated at the level of CYP11B1. However, high sumGC does not necessarily mean sufficient GC action at the time of stress because urinary GCs mainly represented inactivated metabolites. Although maturation of GC metabolizing enzyme activities occurred over time, persisting differences between PT and FT infants in GC precursor levels as well as in GC production were observed, including a constantly higher sumGC metabolite level in PT infants during the first year of life.

Several earlier studies, including ours, have shown that the high secretion rate of fetal zone steroids (eg, DHEAS) in PT infants continues to TEA and then rapidly decreases to similar levels as in FT infants (27, 28, 30, 31). In this study, we show that high activity of the fetal adrenal zone was temporally associated with higher rate of GC precursor production in PT infants. However, this was not reflected in the overall GC production estimated by sumGC, which did not change in relation to TEA as 16α-OH-DHEA and GC precursor levels. CYP11B1 activity, estimated by the product to substrate ratio of sumGC to THS, was the rate-limiting step in GC synthesis. Temporal changes of CYP11B1 activity in the GC pathway and 17,20-lyase activity in the fetal zone pathway were reciprocal in PT infants: CYP11B1 activity increased after TEA as high 17,20-lyase activity decreased. Thus, regulation of GC production at the level of CYP11B1 may protect the developing fetus or in this case the PT infant from excessive GC production at the time when the overall adrenal steroid synthesis in the fetal cortex is massive.

Changes in the activity of CYP11B1 in the PT infants in our study are in line with expression of CYP11B1 in fetal adrenals, showing an increase from <30 weeks of PMA to term (5). Our results of lower CYP11B1 activity in the PT than in the FT infants are also in line with previous cross-sectional studies in plasma (25) and urine (32). Hingre et al reported plasma levels of cortisol precursors (17-OHPreg, 17-OHP, 11-deoxycortisol) and cortisol in PT neonates born <30 weeks of PMA before and after ACTH stimulation test performed at 4 days of age. Cortisol precursors were elevated in PT infants, and elevated substrate-to-product ratio of 11-DOC to cortisol suggested decreased activity of CYP11B1 compared to FT infants, whereas the ratio of 17-OHPreg to 17-OHProg was lower than in FT infants, suggesting higher activity of HSD3B2 in PT infants. This latter finding is also in line with our data, indicating that there is no defect in HSD3B activity in PT infants even before TEA. Another interesting finding by Hingre et al was that in opposition to post-ACTH test increase in the ratios of 17-OHPreg to 17-OHProg and 17-OHProg to 11-DOC, the ratio of 11-DOC to cortisol decreased in half (indicating an increase in cortisol production), suggesting that ACTH stimulated CYP11B1 activity in PT infants especially. Kamrath et al (32). reported levels of 17-HP, THS, THE, and pregnanetriolone (metabolite of 21-deoxycortisol, which is a product of CYP11B1) in spot urine samples of PT and FT infants aged from 3 to 161 days. They observed elevated 17-HP levels and elevated THS/THE ratio but decreased pregnanetriolone levels in PT infants and concluded that elevated 17-OHP levels in PT infants are due to their reduced CYP11B1 activity. CYP21A1 activity did not differ between PT and FT in line with our data.

We found that the total secretion of GC metabolites (sumGC) was significantly higher in PT than in FT infants during the whole first year of life, suggesting that there is no lack in production of GCs in PT infants. However, high sumGC in PT infants already from <30 weeks of PMA does not necessarily mean sufficient GC action at the time of stress as urinary GCs mainly represented inactivated metabolites. Therefore, higher inactivation and excretion rate or shorter half-life in circulation due to, for example, lower binding with carrier proteins could result in high urinary metabolite levels but simultaneous inadequate GC action at tissue level. According to previous studies, activity of 11β-HSD2 (inactivating cortisol to cortisone) is increased in fetal tissues and decreases after birth, although precise data in humans are lacking (reviewed in (33)). Our data suggest that this phenomenon continues in PT infants after birth. Cortisone metabolite levels were 2.5 to 4.9 times higher than cortisol metabolite levels until the ratio decreased close to 1 toward the corrected age of 1 year. This could also reflect a gradual increase of 11β-HSD1 activity (mainly activates cortisone to cortisol although it may function bidirectionally) as previously suggested by changes in plasma cortisol/cortisone ratio during the first year of life (45). High inactivation rate of active GCs might at least to some extent explain the clinical picture of relative adrenal insufficiency in PT infants (46-48). FT infants had very high cortisol metabolite (especially 5α-THF) levels transiently at 1 week of age, which was not seen in PT infants. This suggests transient high activity of HSD11B1 and SRD5A. The role, origin, or mechanism of these high GC levels cannot be explained by our study, but it could be associated with the stress of birth and transition to extrauterine environment. Rogers et al studied 24 hours urinary steroid profiles in healthy FT infants longitudinally during the first year of life (49). Although most GC metabolites showed similar temporal changes as in our cohort, they did not observe a similar peak at 1 week of age in cortisol and 5α-THF as in our FT cohort. Possible explanation could be differences in the sampling time because in our cohort some samples were obtained already at 4 days of age and by 1 week of age, possible changes related to birth may have already disappeared.

Role of 5α-reduction in GC metabolism is to inactivate cortisol for excretion, whereas for androgens 5α-reduction results in formation of a more potent metabolite. SRD5A activity in cortisol metabolism was very low in PT infants before TEA but increased significantly in the first months after TEA to similar levels as in the FT group. This is in line with previous studies reporting that SRD5A1 is not expressed in the fetal liver and its expression increases postnatally (reviewed in (34)). Therefore, using 5α-reduced metabolites in calculations of enzyme activities before TEA should be used cautiously, and changing levels even after TEA (in both PT and FT infants) should be considered. The role of low SRD5A activity in cortisol metabolism in PT infants before TEA is not clear.

Longitudinal study design covering the period from very PT birth to TEA and the first year of life is a strength of our study. Another strength is comparison with longitudinal data of FT infants originating from the same laboratory gas chromatography-mass spectrometry method. Limitations are small size and heterogeneity of the PT infant group. However, they represent a typical cohort of PT infants born <30 weeks of gestational age. Half of the PT infants in our study had received postnatal GC therapy and 8.4% of samples were obtained during the treatment, which might have affected their HPA axis function and GC metabolite levels in urine, but this was considered in the statistical analyses. Due to the difficulty of collecting 24-hour samples from the PT infants, spot urine samples were used instead. Same method was used in both PT and FT cohorts, so it does not explain the differences between the groups.

Both animal and human studies demonstrate that stressful perinatal events can induce persistent alterations in HPA axis function later in life (reviewed in (13, 50-54)). PT birth disrupts the intra-uterine maturation of the HPA axis and stress-related neural circuits (54). In addition to prenatal stressors, such as chorioamnionitis or placental insufficiency, PT birth is commonly associated with substantial postnatal stress related to physiological immaturity, therapeutic interventions and stressful environment in the neonatal intensive care unit, and possible challenges in early mother-infant attachment (reviewed in (54)). Exposure to systemic GCs during the pre- and postnatal periods, while often essential for survival in extremely PT infants, may influence HPA-axis development and have longstanding effects on neurobehavioral outcomes, psychopathology, and cardiometabolic health (13, 14, 26, 53, 54). In humans, perinatal adversity has been associated with both hyper- and hypoactivity of HPA axis, with evidence suggesting that these alterations may evolve across developmental stages (50). Stress and exposure to exogenous GCs may affect the regulation of key HPA axis-related genes, including the glucocorticoid receptor (NR3A1) and HSD11B2 genes, by epigenetic mechanisms (reviewed in (50, 52-54)). Such epigenetic modifications following PT birth have been implicated in altered neurobehavioral trajectories as well as cardiovascular and endocrine function (55, 56). Although robust evidence links PT birth with an increased risk of later metabolic syndrome characterized by insulin resistance, central obesity, hypertension, and dyslipidemia (57), the contribution of early endogenous or exogenous GC exposure to metabolic programming in individuals born PT remains incompletely understood. Our findings provide insight into developmental changes in GC metabolism over time, including during the critical period corresponding to the final trimester of gestation. These data may help elucidate factors that increase susceptibility to long-term programming effects in this population. Further research is required to determine whether the differences observed in GC precursor and metabolite levels between PT and FT infants during the first year of life already signal specific programming of their HPA axis.

In summary, longitudinal data obtained in this study, complemented with comparisons to FT infants, provide insight into maturation of the GC biosynthetic pathway and suggest that both the fetal zone pathway and the GC pathway are subject to developmental regulation in PT infants. Levels of GC precursors upstream of CYP11B1 remain elevated until TEA. Nevertheless, overall GC production in PT infants born before 30 weeks of gestation appears to be maintained and not compromised prior to TEA, consistently remaining at or above levels observed in FT infants. However, high rate of tissue-level GC inactivation may limit the biological effectiveness of circulating GCs. Future studies integrating detailed clinical data with longitudinal serum and urinary GC metabolomic profiling together with epigenetic analyses may enable a more comprehensive understanding of the physiological significance of altered GC biosynthesis following extremely PT birth and its possible contribution to relative adrenal insufficiency and the development of BPD.

## Data Availability

All datasets generated during and/or analyzed during the current study are not publicly available but are available from the corresponding author on reasonable request.

## Abbreviations

5-PT: pregnanetriol
5α-THF: 5α-tetrahydrocortisol
17-HP: 17-hydroxypregnanolone
17-OHP: 17-hydroxyprogesterone
17-OHPreg: 17-hydroxypregnenolone
BPD: bronchopulmonary dysplasia
CA: corrected age
DHEAS: dehydroepiandrosterone sulfate
FT: full term
GC: glucocorticoid
HPA: hypothalamic-pituitary-adrenal
PD: pregnanediol
PMA: postmenstrual age
PT: preterm
sumGC: sum of GC metabolites
TEA: term-equivalent age
THF: tetrahydro-11-deoxycortisol
THS: tetrahydro-11-deoxycortisol
